# Personalized Nutrition as a Key Contributor to Improving Radiation Response in Breast Cancer

**DOI:** 10.3390/ijms23010175

**Published:** 2021-12-24

**Authors:** Anuradha A. Shastri, Joseph Lombardo, Samantha C. Okere, Stephanie Higgins, Brittany C. Smith, Tiziana DeAngelis, Ajay Palagani, Kamryn Hines, Daniel A. Monti, Stella Volpe, Edith P. Mitchell, Nicole L. Simone

**Affiliations:** 1 Department of Radiation Oncology, Sidney Kimmel Cancer Center, Thomas Jefferson University, Philadelphia, PA 19107, USA; axs791@jefferson.edu (A.A.S.); Joseph.Lombardo@jefferson.edu (J.L.); Samantha.Okere@students.jefferson.edu (S.C.O.); Stephanie.Higgins@students.jefferson.edu (S.H.); bs252869@pcom.edu (B.C.S.); Tiziana.DeAngelis@jefferson.edu (T.D.); ajaypalagani@gmail.com (A.P.); Kamryn.Hines@jefferson.edu (K.H.); 2 Department of Integrative Medicine and Nutritional Sciences, Marcus Institute of Integrative Health, Thomas Jefferson University, Philadelphia, PA 19107, USA; daniel.monti@jefferson.edu; 3 Department of Human Nutrition, Foods and Exercise, College of Agriculture and Life Sciences, Virginia Tech, Blacksburg, VA 24061, USA; stellalv@vt.edu; 4 Department of Medical Oncology, Sidney Kimmel Cancer Center, Thomas Jefferson University, Philadelphia, PA 19107, USA; edith.mitchell@jefferson.edu

**Keywords:** breast cancer, radiation therapy, nutrient, African American, obesity, metabolic syndrome, aging

## Abstract

Understanding metabolic and immune regulation inherent to patient populations is key to improving the radiation response for our patients. To date, radiation therapy regimens are prescribed based on tumor type and stage. Patient populations who are noted to have a poor response to radiation such as those of African American descent, those who have obesity or metabolic syndrome, or senior adult oncology patients, should be considered for concurrent therapies with radiation that will improve response. Here, we explore these populations of breast cancer patients, who frequently display radiation resistance and increased mortality rates, and identify the molecular underpinnings that are, in part, responsible for the radiation response and that result in an immune-suppressive tumor microenvironment. The resulting immune phenotype is discussed to understand how antitumor immunity could be improved. Correcting nutrient deficiencies observed in these populations should be considered as a means to improve the therapeutic index of radiation therapy.

## 1. Introduction

To date, radiation regimens for breast cancer are chosen and administered based on a patient’s tumor type and stage; typically, the only aspect of a radiation regimen that is changed is dosing, fractionation, overall treatment time, and volume of the breast and normal tissue treated. This approach works for a majority of patients since local recurrence rates are low for most breast cancer [1,2]. Since there is evidence that optimal local tumor control portends improved survival and fewer metastases, attention should turn toward differentiating which tumor types or patient characteristics might be associated with poor outcomes despite adequate radiation therapy (RT). It has been established that certain patient characteristics or underlying tumor genetic milieu are associated with varying degrees of radiation sensitivity and these are not accounted for [3]. Despite this finding, to date, there are scarce data for combining radiation with systemic therapies including chemotherapy or immunotherapy to improve the effect of radiation.

After the explosion of the understanding of genomic mutations and technology approaches for sequencing, the last two decades have been met with precision medicine approaches to understanding how tumors are growing and progressing [4]. Although chemotherapy and other systemic treatments have been tailored by genomic alterations for some women with breast cancer, successful modifications to radiation therapy regimens have been mostly limited to changes in the number of fractions or doses given. Genetic signatures of a patient’s tumor have been used to determine if a breast cancer patient would benefit from chemotherapy. Genomic profiling of tumors has also been used to guide treatment decisions for metastatic breast cancer patients and prostate cancers of various risks. Unfortunately, few approaches to genomic profiling have been devised to assess radiation response with the most notable including the radiation sensitivity index [5]. To our knowledge, genomic indicators of radiation response have not yet been used to alter radiation regimens. Despite the increase in precision medicine approaches, significant, improved cancer outcomes have not followed.

Simultaneously, the last two decades have also brought a change in the United States patient population without adaptation of radiation based on the characteristics of our population. Patients with obesity, metabolic syndrome, advanced age, and diverse communities have increased with time and are known to have varying levels of radiation sensitivity [6,7,8]. This is likely, in part, due to alteration of specific molecular pathways that are associated with a decrease in antitumor immunity, which can influence tumor biology and response to radiation. Additionally, these patient populations often have specific nutritional deficiencies that further dysregulate metabolic pathways and immune response. Understanding nutritional deficiencies in various populations of patients may provide insight into nontoxic methods to increase radiation responsiveness. Precision nutrition, defined as an approach that is tailored based on hosts genetic, phenotypic, microbiome profiles, and medical history [9,10], has long been used to treat nutrient deficiency based diseases including scurvy, anemia, osteoporosis, etc., and micronutrient deficiencies have also been shown to trigger DNA damage and increase risk of cancers [11]. Recent studies have shown the benefit of applying precision nutrition to target specific metabolic pathways and preventing cancer relapse in multiple cancers including breast cancer [12,13]. Precision nutrition has also been shown to modulate the gut microbiome, foods rich in phytochemicals and omega-3 fats have been shown to alter the microbiome and increase the abundance of anti-inflammatory bacteria that help prevent cancer progression [14,15]. In the current narrative review, we discuss populations with known differences in radiation outcome—African Americans, obese patients or those with metabolic syndrome, and the aging population. We then explore probable mechanisms by which precision nutrition interventions could be used to improve radiation response in nutrient-deficient resistant breast cancer patients, leading to enhanced tumor control and/or less radiation toxicity while decreasing the inequity of outcomes.

We propose that improving radiation response can only be accomplished if a patient’s baseline characteristics, which are observed to have associated alteration in metabolic and immune function, are accounted for as these directly influence the tumor and the tumor’s response to therapy. To identify ways to personalize radiation, here, we discuss specific patient populations known to be associated with poor radiation response and their associated molecular underpinnings, and we identify precision nutrition approaches to improving radiation sensitivity to improve cancer outcomes for all patients. The future of radiation oncology, moving toward precision radiation, will need to account for molecular underpinnings specific to host populations and implement combination therapy strategies to even the playing field for all patients receiving radiation.

## 2. Breast Cancer and Radiation Response in the African American Population

From 2008 to 2012, the incidence of breast cancer has increased among the United States African American (AA) population, as per the American Cancer Society [16]. There is also a mortality disparity with African American women, having a 42% higher mortality rate than Caucasian women nationwide [16]. Unfortunately, it has also been shown that the mortality increase for African American women holds for all breast cancer subtypes, including estrogen positive, her-2-neu positive, and triple-negative breast cancers [17]. The cause for the observed disparity is multifactorial and includes differences in genetics, a socioeconomic status that often delayed access to care and treatment, and toxicities due to treatment that weakens the overall prognosis [18].

### 2.1. Molecular Disparity in African American Breast Cancer Patients

It is established that some of the disparity in breast cancer outcomes may be related to the more notable dysregulation of the IGF-1R pathway in African American patients. African American patients suffer disproportionally, compared with their Caucasian peers, with metabolic problems that affect the IGF-1R pathway. Comorbidities such as diabetes, abdominal obesity, dyslipidemia, glucose intolerance, and metabolic syndrome (MetS) are higher in the African American community and lead to upregulation of the IGF-1R signaling pathway [19]. IGF-1R is expressed significantly more in African American normal tissue, compared with that of Caucasian patients [20], and specifically more in the breast tissue [21], with increased dysregulation in cancers.

In breast cancer, metabolism is, in part, responsible for metastatic spread via dysregulation of the IGF-1R/Akt pathway, which directly influences tumor progression and plays a role in the fate of anchoring metastases to the tumor microenvironment [22,23,24]. IGF-1R overexpression is associated with decreased breast cancer survival, increases in recurrence, and treatment resistance to radiation and Herceptin, which are both used to treat brain metastases [25,26,27]. In addition, IGF-1 and IGF binding protein 3 are also associated with breast cancer risk, progression, recurrence, and the probability of survival in African American women [28,29]. Therapies that can be added to radiation that increase local control and metastases would be optimal.

### 2.2. IGF-1R and Radiation Response

Upregulation of IGF-1R, which is notable in the African American community, is also associated with increased radiation resistance due to decreases in apoptosis and antitumor immunity [30,31,32].

The influence of IGF-1R signaling after radiation may influence cancer cell survival due to alterations in apoptotic response. The mechanism by which IGF-1R is believed to cause resistance to radiation therapy and increase cell survival is by acting on the BCL2/BAD complex to inhibit BAD through phosphorylation, thereby releasing antiapoptotic BCL-2, and preventing apoptosis. IGF-1R also curbs apoptosis in coordination with major vault protein (MVP), which itself inhibits PTEN and increases inhibition of apoptosis through activation of PI3K/Akt [33]. When the apoptosis mechanism is not optimally functional, the DNA damage induced by radiation therapy that is left unrepaired is unable to be followed by apoptosis. Further, a combination of overexpression of IGF-1R and radiation-induced non-homologous end joining (NHEJ) leads to downregulation of apoptosis and thus radiation resistance [33,34].

IGF-1R upregulation also directly creates an immune-suppressive environment that further accentuates radiation resistance with a decrease in antitumor immunity via CD8+ T cells and M1 macrophages and an increase in regulator T cells and M2 macrophages. In in vivo breast cancer models, the inhibition of IGF-1R reduced tumor growth and increased the CD8+-mediated immune response in the tumor while reducing immunosuppressive regulatory T cells. Although African American women with breast cancer are noted to have higher levels of CD8+ T cells, as noted in a study of 688 invasive breast cancer tumor samples (550 Black and 138 White), it has been shown that the high CD8+ proportion does not translate to a better prognosis in AA women, because the CD8+ population is made up of a higher percentage of exhausted CD8+ T cells, which is linked to lower survival [35,36].

Similarly, while CD8+ T cells are reported to be a marker for favorable prognosis, immunosuppressive regulatory T cell (Treg) infiltrates are associated with distant metastases, worse survival [37], and radiation resistance [38]. In fact, Treg ablation has been found to significantly improve ionizing radiation therapy [39]. Unfortunately, Tregs have consistently been shown to be higher in AA patients, compared with CC patients [40]. Tumor-associated macrophages are known to secrete IGF-1/IGF-2 in the tumor microenvironment. The activation of the IGF-1R/Akt pathway triggers increased infiltration of the M2 macrophages, leading to tumor progression and decreased overall survival in breast cancer patients. AA women have increased proliferation of immunosuppressive M2 macrophages, as compared with M1 macrophages, which may inhibit CD8+ action [41]. “M2-shifted” groups are strongly associated with decreased disease free-survival, lending to the idea that standard BCT alone may be insufficient for these patients [41,42]. Tumor-associated M2 macrophages suppress postradiation therapy antitumor immunity, which promotes tumor regrowth and angiogenesis [43,44,45] and radiation therapy resistance [46,47].

Ultimately, the increase in the IGF-1R signaling pathway leads to tumor invasion and metastasis with evasion from the immune system, which contributes to radiation resistance and poor breast cancer outcomes in AA patients [48]. This may be, in part, due to increased rates of obesity in African American women, which is often associated with insulin resistance and dysfunction of IGF-1R [49,50]. Taken together, standard radiation regimens may not be sufficient for AA patients with breast cancer due to the underlying molecular alterations in the IGF-1R signaling pathway.

### 2.3. Vitamin D Supplementation and IGF-1R to Improve Radiation Response

Since African American breast cancer patients have upregulation of the IGF-1R signaling pathway, which is associated with poor radiation response, identifying a mechanism to downregulate the pathway could optimize radiation response. Interestingly, it is well known that vitamin D can downregulate the IGF-1R signaling pathway. In vitro studies carried out on breast cancer cells showed that vitamin D treatment can downregulate the IGF-1R pathway and increase apoptosis [51]. Vitamin D and its analogs have also been shown to suppress IGF-1-induced growth of breast cancer cells by downregulating IGF and IGF-1R and increasing IGF-BP expression [52]. In addition, most African American women have a vitamin D deficiency. Here, we explore the link and show preliminary data demonstrating the possible benefit of vitamin D in regulating radiation response.

### 2.4. Vitamin D Deficiency in AA Patients

Vitamin D deficiency is a common presentation for African American patients. This can be attributed to several factors including darker skin pigmentation, low dietary vitamin D, and obesity. Vitamin D synthesis from sun exposure to skin provides 50–90% of vitamin D in the human body, the remainder comes from dietary and supplementary intake [53]. The concentration of melanin in the skin of the AA population dramatically suppresses the cutaneous synthesis of vitamin D by preventing the penetration of UVB light. While this is beneficial when living in areas of intense sunlight such as equatorial Africa, or more tropical climates where that protection is needed while still allowing for adequate vitamin D production, dark skin pigmentation puts African Americans living in non-tropical areas at higher risk of vitamin D deficiency. Additionally, since vitamin D is a fat-soluble vitamin, it may be sequestered by adipose tissue, which lowers physiologically availability in circulation, and unfortunately, the African American population in our country has higher rates of obesity, compared with European Americans [54].

### 2.5. Vitamin D and Breast Cancer

Multiple studies demonstrate that low vitamin D levels correlate to the risk of breast cancer and worse breast cancer outcomes. Vitamin D deficiency has been shown to increase breast cancer susceptibility by approximately 23% in AA women, indicating vitamin D intake could be considered a preventative factor for breast cancer incidence [55]. Higher vitamin D intake has been linked with decreased breast density, which is associated with decreased risk of breast cancer [56]. Increased overall survival, especially in premenopausal women, was associated with an elevation in serum 25 (OH)D concentrations in a recent cohort study that included 1666 women diagnosed with breast cancer [55]. The mechanism by which vitamin D may prevent breast cancer and lead to improved cancer outcomes may be, in part, due to the downregulation of the IGF-1R pathway.

### 2.6. Immune Response and Radiation Response

Vitamin D may help address the immune dysfunction associated with IGF-1R dysregulation seen in AA breast cancer patients. In the presence of vitamin D, breast tumors showed an increase in tumor-infiltrating CD8+ T cells that were functionally active. Interestingly, a high-fat diet was shown to reverse this vitamin-D-induced increase in tumor-infiltrating CD8+ T cells, highlighting the importance of diet in tumor growth [57]. Vitamin D has also been shown to induce Foxp3+ Treg cells, making them less immunosuppressive [58].

As we have discussed above, AA patients have a higher incidence of vitamin D deficiency, and vitamin D has shown some promise in breast cancer incidence and outcomes [54]. Diet is a major contributor to health disparity in breast cancer and other chronic diseases. A person’s diet can increase or decrease his or her risk for cancer. Nutritional factors including dietary fat, meat, fiber, and vitamin D have been investigated as either promoting or inhibiting breast cancer development and survival [59]. The active form of vitamin D has been shown to efficiently contribute to increased genomic instability in response to radiation [60].

### 2.7. Vitamin D Supplementation to Improve Radiation Response in African American Women

**Figure 1:**
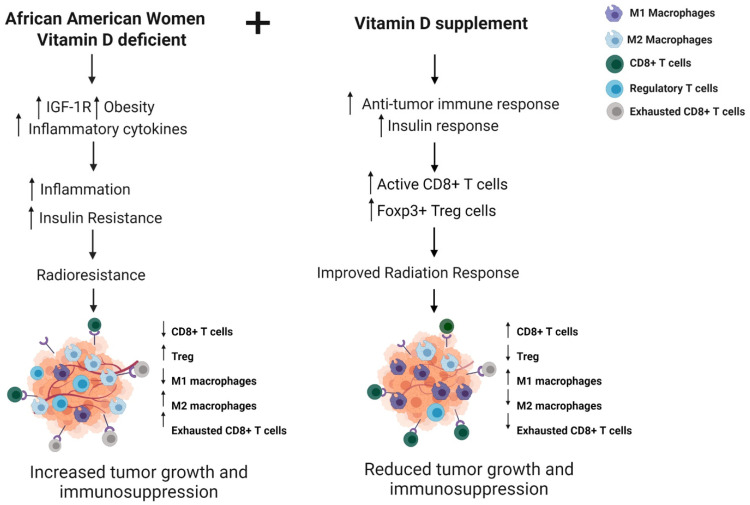
Vitamin D supplementation to modulate antitumor immune response and radiation sensitivity in African American breast cancer patients. Vitamin D deficiency is prevalent in African American women and is associated with upregulation of the IGF-1R signaling pathway with resulting immunosuppression and increase in inflammatory cytokine levels leading to tumor growth and radiation resistance. Modeling supplementation with vitamin D, radiation response would improve by increasing antitumor immune response. Created with BioRender.com.

In Figure 1, we summarize the factors responsible for the poor radiation response observed in African American women and hypothesize that supplementation with vitamin D will improve the radiation response to tumors in this population. We propose that the mechanism by which vitamin D will improve tumor response is by increasing antitumor immunity and insulin response, thereby preventing inflammation-related radioresistance.

## 3. Obesity, Metabolic Syndrome, and RT

Breast cancer patients who are obese or have metabolic syndrome have an increased risk of breast cancer, worse cancer outcomes, and radiation resistance [61]. According to the CDC, the prevalence of obesity among adults in the United States is 42.5%, with 9.2% being severely obese [62]. It has been found that metabolic syndrome leads to a 47% increase in relative risk of breast cancer [63], which spans across all breast cancer subtypes [64,65].

Mechanistically, patients who are obese or have metabolic syndrome commonly have insulin resistance and an adipokine imbalance, which results in systemic inflammation that is known to be associated with disease progression and work outcomes.

The insulin resistance results in increased production of IGF-1, resulting in upregulation of the IGF-1R/AKT pathway, promoting carcinogenesis, angiogenesis, migration, and invasion. IGF-1R also inhibits apoptosis [66,67].

The adipokine imbalance is notable for a decrease in levels of adiponectin, with increased leptin levels. This combination negatively regulates inflammation and cell proliferation, leading to increased breast cancer risk [68,69], cell proliferation, migration, and invasion [70]. Unfortunately, the adipokine imbalance plays a role in proinflammatory pathways, resulting in the release of TNF-α, IL-1, IL-6, and IL-12 and reactive oxygen species (ROS) [68]. Systemic inflammation due to obesity/metabolic syndrome results in lipolysis, which releases free fatty acids (FFAs). FFAs stimulate TLR4 present on breast cancer cells, activating NF-kB, which increases cancer stem cells. The inflammatory cytokines, including IL-8, IL-6, CCL2, CCL5, and IP-10 produced from adipocytes, promote cancer stem cell expansion.

In the setting of obesity and insulin resistance, immune dysfunction is prevalent. The tumor microenvironment in obese patients has increased Tregs, exhausted CD8+ T cells, and increased M2 macrophages. Tumor cells in the setting of obesity are stimulated by inflammatory cytokines and make CCL22, which recruits Tregs that have an inhibitory effect on the antitumor function of CD8+ T cells [18]. A study in obese mice showed that the mice produced high amounts of leptin that activated STAT3 in CD8+ T cells, promoting fatty acid oxidation. Fatty acid oxidation in CD8+ T-effector cells decreased their antitumor function, resulting in T cells with an exhausted phenotype [71], thereby promoting breast tumorigenesis [72]. Tissue exposed to inflammatory cytokines, as with obesity, switches from M1 to M2 macrophages [73], which produce epithelial growth factor (EGF) and tumor growth factor-beta (TGF-β), thereby promoting tumor invasion and metastasis [50].

### 3.1. Obesity/Metabolic Syndrome and Radiation Response and Toxicity

Prior studies have demonstrated that obesity and associated insulin resistance portends for poor outcomes and radiation response. A recently published large meta-analysis of over 200 studies shows obesity was associated with decreased overall survival (HR, 1.14; 95% CI, 1.09–1.19; *p* < 0.001 as well as cancer-specific survival, *p* < 0.001 [74]. Specifically, in breast cancer, obese patients have up to a 40% increased risk of breast cancer recurrence [75]. Obese women are also known to have a greater incidence of metastatic disease from breast cancer [76]. For women with diabetes who develop breast cancer, population-based studies have shown that even with similar cancer treatment, women with an extended history of diabetes had a higher all-cause and breast-cancer-specific mortality [77].

Preclinical studies have linked obesity and insulin resistance as seen in metabolic syndrome with cancer progression through immune evasion and radiation resistance [6]. Obese breast cancer patients who received whole-breast radiation were 12.6% more likely to have local recurrence after five years. Kim et al. showed that adipose stem cells, together with leptin in the tumor microenvironment, were responsible for the obesity-associated radiation resistance through upregulation of NOTCH and IL-6 [78]. In addition to the understanding that obesity and metabolic syndrome impact outcomes at initial diagnosis, it has also been demonstrated that obesity and diabetes impact outcomes for metastatic patients. Patients with breast cancer brain metastases who were either obese or diabetic and were treated with whole-brain radiation showed decreased overall and progression-free survival [6].

Obese patients have been shown to suffer from worse radiation-induced toxicities than patients with normal BMI. Obese breast cancer patients show an increase in inflammatory biomarkers due to radiation causing skin toxicity. Obese patients have increased pain, worse functional well-being during treatment, and slower improvement [79]. An increase in inflammatory biomarker CRP is also seen, which is linked to the radiation-induced early adverse skin reactions (EASR). Obesity-related increase in inflammation increases EASR and causes changes in proinflammatory, proangiogenic, profibrotic cytokines resulting in increased normal tissue toxicity [80]. High BMI in breast cancer patients has also been linked to worse acute treatment outcomes and lower quality of life pre, during, and postradiation treatment. Obesity is also linked to a worse FACT g score and physical well-being score. Patients with high BMI reported worse symptoms including fatigue, drowsiness, shortness of breath, and pain [81]. Higher BMI is also associated with an increase in the development of radiation dermatitis after radiation therapy following breast-conserving surgery. Larger breast size has also been associated with increased toxicity due to higher dosage and dose homogeneity [82].

### 3.2. Magnesium in Obesity/Metabolic Syndrome and Breast Cancer

Hypomagnesemia is commonly observed in patients with metabolic syndrome and obesity. Magnesium plays a role in glucose metabolism and insulin signaling, regulating tyrosine kinase activity and glucose transporter protein activity 4 (GLUT4), leading to glucose translocation into the cell [83]. In their study with metabolic syndrome patients, Guerrero-Romero et al. showed that hypomagnesemia was linked with dyslipidemia, hypertension, and insulin resistance. Magnesium supplementation improved insulin sensitivity [84]. Hruby et al. showed that, compared with the control group, participants with the highest magnesium intake had a 47% reduced risk of metabolic disorder or diabetes incidence and had lowered fasting glucose and insulin resistance [85]. Volpe suggests that if magnesium supplementation affects insulin sensitivity in participants with diabetes mellitus, it may also improve insulin sensitivity in obese individuals at risk of type 2 diabetes mellitus [83]. Supplementation with magnesium in overweight subjects with insulin resistance for six months resulted in a significant difference in fasting glucose and insulin sensitivity [86]. Similarly, another study showed that higher dietary magnesium intake was strongly associated with the attenuation of insulin resistance and was more beneficial for overweight and obese individuals in the general population and premenopausal women [87].

Magnesium deficiency has been shown to both protect against and increase the risk of breast cancer. It plays a vital role in the cell cycle, and hence, its deficiency influences precancerous transformation [88]. Magnesium is also involved in acquiring immunocompetence and increased levels indicate protection from cancer [89]. Huang et al. have shown that breast cancer risk was reduced in those with higher intake of magnesium through a direct effect on breast cancer and an indirect effect by reducing CRP levels [90]. Low intracellular magnesium levels at the beginning of tumorigenesis are associated with cancer development and progression due to impaired antioxidant defense and increased inflammation [91]. Low magnesium levels also induce nitric oxide production, leading to the production of VEGF and angiogenesis, which leads to an increase in angiogenesis [92]. Contradictory studies have shown that high magnesium levels can stimulate breast cancer development at the early stages of tumorigenesis by regulating enzymes and genes that trigger energy generation and inhibit apoptosis [93,94,95,96].

### 3.3. Magnesium Supplementation to Improve Radiation Response

**Figure 2:**
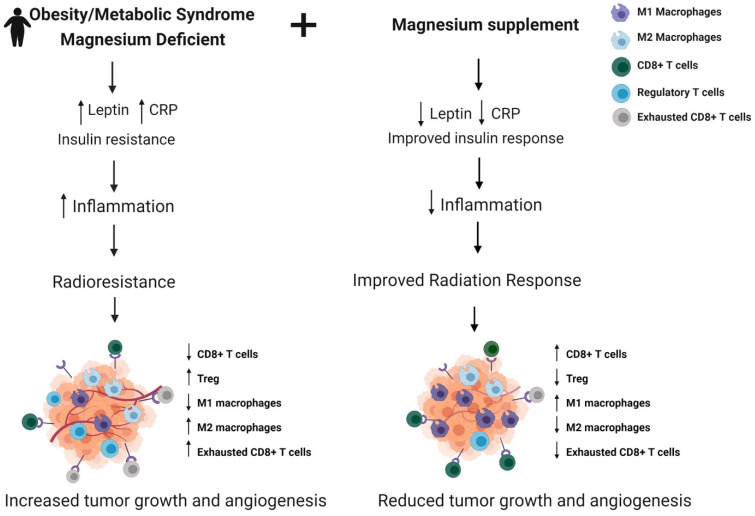
Magnesium supplementation to modulate antitumor immune response and radiation sensitivity in breast cancer patients with obesity/metabolic syndrome. Magnesium deficiency is commonly observed in individuals with obesity and metabolic syndrome and is associated with increased insulin resistance and inflammation in these patients, leading to radiation resistance and tumor growth. Modeling supplementation with Magnesium, radiation response would be improved by increasing insulin sensitivity and antitumor immune response. Created with BioRender.com.

In Figure 2, we summarize the factor responsible for the poor radiation response that is observed in obese breast cancer patients or those with metabolic syndrome. Since magnesium is involved in the regulation of insulin signaling, leptin metabolism, and the immune response, we hypothesize that supplementation with magnesium in this population would help increase the efficiency of radiation therapy and tumor response by reducing levels of inflammatory markers and improving insulin response, thereby preventing tumor progression, reducing toxicities, and improving breast cancer outcomes in this population.

## 4. Breast Cancer and Radiation Response in Aged Population

### 4.1. Aging and Breast Cancer: Influence of Immune Dysfunction

The vast majority of cancers are associated with aging and the incidence increases drastically with chronologic age. Currently, the aptly named “silver tsunami”, or a rise in the aging population, will bring a shift in the cancer patient population with approximately half of all cancers diagnosed in adults aged ≥65. However, it is believed that in less than 10 years, 70% of all cancer will occur among those 65 and older [97]. A recent SEER analysis estimates that due to the increasing aging population, the incidence of invasive breast cancer cases could double by 2030, and women older than 70 will make an increasing proportion of those cases [98].

Understanding the reasons behind the increase in cancer incidence in this population at a cellular level is challenging. It has become apparent that older adults have metabolic dysfunctions that may contribute to the development of cancer as well as influence cancer treatment outcomes. As it pertains to aging, possible realistic explanations for the association with increased breast cancer incidence, pathogenies, and tumor progression include the relationship between age-related inflammation, DNA damage, immune cell senescence, and increased adipose with changes seen in metabolic and immune function. As humans age, there are changes in metabolic function with an intimate interplay with the immune system. With age, there is an accumulation of senescent cells or cells that have lost their ability to proliferate. These cells occur throughout life; however, the ability to clear them decreases with aging. Interestingly, even with the cells losing their ability to proliferate, they still have high metabolic activity [99]. There is a metabolic change that occurs shifting toward glycolytic metabolism even with oxygen present. Shifts in metabolism increase AMP and ADP and activate AMPK. This so-called aerobic glycolysis is known as the Warburg Effect and has been shown to promote tumorigenesis and cancer progression [100]. Studies have shown some specific metabolic changes that occur in immune cells with aging. For example, there is a metabolic difference in elderly T cells with these cells having a lack of substrate for mitochondrial respiration. In this circumstance, they enter the pentose phosphate pathway and an anabolic state [101]. This accumulates NADPH, and there is an upregulation of AMPK, targeting dual-specificity protein phosphatase, which, in turn, regulates MAPK. Overall, this process leads to a decrease in proper T-cell function, leading to a more protumor environment.

The decline in immune function, particularly the adaptive immune response, with aging is known as immunosenescence. The overall lymphoid numbers decrease, with the greatest change in function observed in T cells [102]. Aging causes increased proinflammatory memory T cells and decreases the number of naïve T cells. This whole process is likely linked to the chronic inflammation seen in aging, or “inflammaging” [103]. In breast cancer, several studies have correlated “inflammaging” with changes in micro-RNA and chronic inflammation. These non-coding RNAs regulate gene expression and have been shown to influence the inflammatory state seen in the elderly, for example, increased levels of microRNA-21 have been observed in invasive breast cancers [104]. A recent study of breast cancer patients found aging was linked to lower levels of lymphocyte infiltration and decreased CD8 cells. The researchers ultimately compared these observed changes with clinical frailty and found a correlation between immune changes and frailty [105]. Specifically, in breast cancer, there is evidence that this inflammation-shifted immune system influences the increased risk of breast cancer and worse prognosis in this aging patient population. [106] Another pathway where aging metabolism affects immune response can be exemplified by the age-related changes in adipose accumulation, which is also associated with an immune-suppressive environment.

### 4.2. Radiation Therapy Outcomes and Toxicity in Older Patients

While outcomes in the elderly population could be impacted by a diagnosis at a later stage of disease or undertreatment, it is likely age-associated changes in metabolism, immune function, and inflammation play a role [107,108]. Understanding the biological process that truly underlies aging as it relates to cancer and the ability to tolerate cancer treatment will be the next large step in individualizing care in this important and growing geriatric population [109].

In addition to biological factors, there are socioeconomic factors that may contribute. While poverty is linked with worse breast cancer outcomes in general, roughly 1 in 3 adults older than 65 in the United States are economically insecure [110]. More older women live in poverty, with a potential explanation being wage discrimination and time away from work to raise kids. Economic inequity can lead to worse nutrition, poor access to food, and a lack of education on healthy lifestyle choices. There is an increased number of comorbidities including obesity and diabetes. The International Agency for Research on Cancer reported up to a third of all cancer cases are linked with increased weight and lack of physical activity [111,112]. It has been shown that obesity and poor nutrition accelerates premature aging, and it stands to reason that the biological explanation for increased cancer and worse outcomes associated with some of these factors is related to the changes in cellular metabolism and immune function previously described.

In oncology, there has been growing interest in predicting and improving the difference in cancer treatment outcomes and side effects seen in geriatric patients. There exist validated tools to help predict chemotherapy toxicity in this population. Unfortunately, the data regarding outcomes and side effects of radiation in the aging population are sparse. It is known, however, that our older adult population has increasing difficulty with radiation-induced fatigue, which is more pronounced in more frail patients [113].

### 4.3. Zinc Supplementation to Improve Radiation Response

As life expectancy increases, we will need to continue to research novel approaches to optimize radiation response despite changes in metabolism and immune function in our aging population.

As the medical community continues to learn about the interplay between aging, metabolism, and immune function as it relates to cancer, solutions such as a precision nutritional approach with nutrients such as zinc to narrow the health disparity discussed may be feasible [114]. Even minor deficiencies in Zinc can have large cellular effects [115,116]. Zinc deficiency is associated with increased breast cancer incidence and disease progression [117]. Higher zinc levels have been linked with decreases in breast cancer, and in animal models, zinc supplementation has been shown to decrease breast cancer. High levels of zinc supplementation had a positive effect on reducing oxidative stress and improving immune responses in cancer patients and can also improve response to radiation [118,119].

Zinc levels also have implications on immune function, which may explain the improved breast cancer outcomes and response to radiation. These findings may be related to the effect, or lack thereof, that zinc has on metabolic and immune function with aging. For the first time, in their in vitro study, Dierichs et al. showed that zinc supplementation is able to influence the polarization of human-derived macrophages. They used HLA-DR as an M1 marker and Dectin-1 for M2. Zinc supplementation via high extracellular Zn^2+^ resulted in increased HLA-DR and decreased Dectin-1, signifying that Zinc promoted M1 polarization while decreasing M2 [120]. Clinically, eliminating macrophages in tumor cells and the microenvironment is a large challenge. One approach may be to change the polarization from protumor to antitumor using agents such as zinc. Other studies have shown zinc deficiency affecting T-cell activation, and a randomized, controlled trial carried out on elderly adults showed that 25 mg of zinc sulfate for 3 months increased the levels of activated T cells [121].

**Figure 3:**
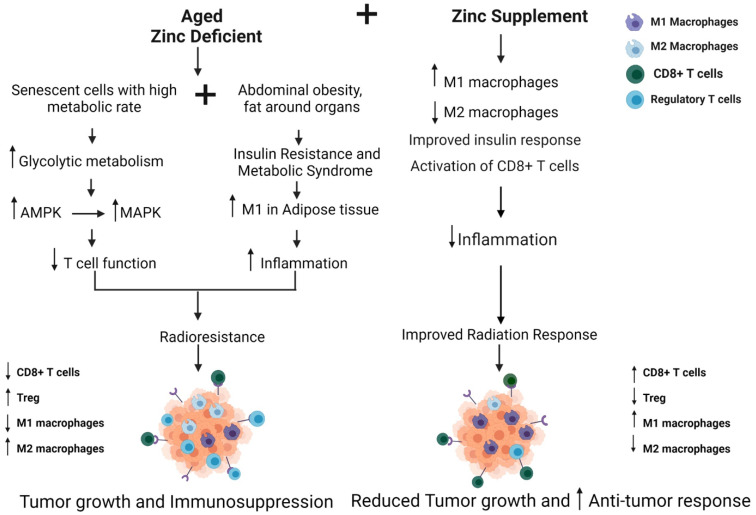
Zinc supplementation to modulate antitumor immune response and radiation sensitivity in the senior adult breast cancer patient. Older women show abdominal obesity and insulin resistance with inflamed adipose tissue and possess senescent cells that show elevated glycolytic metabolism, leading to reduced T-cell function and immunosuppression. Zinc deficiency is commonly observed in older women. Modeling supplementation with Zinc, radiation response would be improved by reducing inflammation and improving insulin and immune response, thereby reducing breast cancer tumor growth [108,117,121]. Created with BioRender.com.

The implications of metabolism and aging immune function are clearly becoming more important as the average population of the United States shifts to an older and likely more obese one. In Figure 3, we summarize the factors that contribute to poor radiation response in the older adult population. We hypothesize that incorporating a targeted treatment approach for the aging population, using zinc supplementation to alter metabolism and immune response, may help in altering the response to radiation for the older adult population with breast cancer.

## 5. Conclusions

Optimizing radiation therapy will ultimately need to account for both tumor characteristics and patient characteristics. The disparity in interpatient tumor response is likely due to metabolic and immune dysregulation of the patients. Understanding patient populations and underlying molecular dysregulation will provide insight into optimizing response to radiation. Specific metabolic states or trends are predictable in patient populations and learning to account for variations will ensure more equitable radiation delivery. Implementing a precision nutrition approach during radiation therapy, with the goal of improving population-specific metabolic disruptions, may allow for the augmentation of radiation therapy. Further clinical trials need to be conducted to test different dietary supplements as enhancers of radiation therapy.

## Data Availability

The data that support the findings of this study are available from the corresponding author upon reasonable request.
